# Abstract of the Proceedings of the Buffalo Medical Association

**Published:** 1859-01

**Authors:** Austin Flint


ART. IV.—*Abstract of Proceedings of the Buffalo Medical Association.*

TUESDAY EVENING, Dec. 7, 1858.

The Association met.

Present—The President, Dr. Wyckoff in the chair; Drs. Miner, Ring, Jansen, Rogers, Rankin, Stevens, Treat, Hutchins, Hawley, Newman, Wilcox, Butler, Strong, White, Gould, Whitney and Boardman.

The minutes of the last meeting were read and approved.

Dr. RANKIN then reported the following case of *Penetrating Wound of the Abdomen:*

On Sunday evening, Sept. 5th, I was called to see a boy named I. L., aged 16 years, who had been stabbed with a common clasp knife. Before my arrival, he had been carried from the street into a house near by; I found him lying upon a bed, laboring under great prostration, dyspnœa, and excessive vomiting. All the symptoms of strangulated hernia were developed.

On searching for wounds, two were found; one upon the top of the middle third of the left thigh, three inches in length, extending through the skin, and fascia, down into the rectus femoris muscle, dividing it through about half its thickness. The other wound was in the abdomen, an inch and three quarters below the umbilicus, three-quarters of an inch to the right of the median line; three quarters of an inch in length, and penetrating into the peritoneal cavity. About an inch and a half of omentum protruded through the opening. I immediately addressed my attention to this wound, first to reduce the hernia; but all attempts at reduction, by traction and compression, were futile, owing partly to the want of a proper instrument to work with, and to the congested and swollen condition in which it was, being in a state of incarceration, with all its blood vessels completely engorged. Under these embarrassing difficulties, and in view of the responsibility of the case, I would not enlarge the wound, and return the omentum, nor resort to the operation of ligation, without council. I therefore requested the friends to send for Dr. L. P. Dayton, and I, in the meantime, devoted my attention to dressing the wound on the thigh. Dr. Dayton arriving, the operation of reducing the hernia of omentum, by the taxis, was again attempted, and successfully terminated, in about twenty minutes, with the use of cold water, steady pressure being kept up on the protruded part, and gently pushing it in with a large spoon probe; the wound was then dressed, with one suture passed through the skin only, adhesive plasters, and a compress, to prevent future rupture, over this a broad roller.

The patient was ordered to remain upon his back, with the chest and hips elevated, so as to produce relaxation of the abdominal muscles, and a grain and a half of opii pulvis given, the dose to be repeated every four hours.

In the course of an hour after the dressing of the wounds, the pulse went down to seventy-five, which, before, was accelerated to over a hundred beats per minute; he soon seemed to rest quite easily, occasionally dozing, and I left for the night.

Monday, Sept. 16, 8 A. M.—Found my patient awake; he had passed a very quiet night; skin cool, respiration good, pulse seventy-five per minute. He seemed so easy, that it was deemed best to remove him home, which was safely accomplished, without the least excitement. Ordered the same treatment as that of last night, with perfect quietude in the supine position, and abstinence from food.

This same treatment was continued for seven days, during which period, there was at no time the least symptom of any peritoneal inflammation, nor even any tenderness over the abdomen; and it seems to me that the free use of opium was the agent which produced so favorable a termination of this case. The morning of the fourth day the suture in the abdomen was removed; the wound had healed kindly by first intention. The compress was continued.

On the morning of the seventh day the opium was discontinued, and three ounces of castor oil given, which operated thoroughly in about four hours, and the patient was permitted to partake of food; from this time he continued to convalesce, and in fourteen days from the receipt of the injury, was about the streets, as well as he ever was.

*Remarks.*—The only interest in this case is, that a serous membrane was wounded, and the result was recovery. Statistics on this subject are meagre, which is to be regretted. Erichsen, Miller, Lizars, Hennen, Liston, Neill and Smith, mention that there are punctured wounds of the abdomen with wounds of the intestines, but merely give the treatment, citing no cases. Chelius (page 402, vol. 1) cites two cases; one, a ramrod shot through the abdomen, sticking into the transverse process of one of the vertebræ, recovered; the other two iron spikes, stuck into the belly, fatal. In these two cases the intestines were uninjured. He also cites four cases, where the intestines were punctured; all died. In Cooper's lectures, two cases are reported. But the best paper on this found in the transactions of the Medical Society of the State of New York, presented by Dr. March. In his closing remarks, after reporting one case, where the intestine and omentum both protruded, he makes use of the following words, which suit this case of mine: "Opium or morphine, in this case, seemed to have the effect to quiet peristaltic action of the bowels, to allay irritation, and thereby to prevent the accession of peritoneal inflammation, objects of the highest importance in the treatment of the case."

Dr. Hawley presented the following case of

### *Abortion between the Fourth and Fifth Months, Supposed to be due to Absorption of the Spirits of Turpentine.*

Mrs. T. in her first pregnancy, between the fourth and fifth months, was employed in rendering engravings transparent, by immersing them in spirits of turpentine. This process required her to pass several hours of the day in a warm room, filled with vapors of turpentine, and to immerse her hands and arms in the fluid. After practising this for several days, she one night found herself obliged to rise often, for the purpose of urination, and observed the water passed to be bloody. This, in her inexperience, she imagined to be uterine hæmorrhage, and a token of approaching miscarriage. She at once sent for me. I found the os uteri undilated, and not a trace of blood in the vagina. On inspection of the water, it was apparent that the blood proceeded from the bladder. She stated that the motions of the child had ceased. The next morning the abdomen was carefully auscultated, but no sound of the fœtal heart could be discovered. The supposition was, that the fœtus was dead, but still no sign of labor appeared. In the meantime the hæmaturia ceased. On the third day after the appearance of the bloody urine, labor came on naturally, and proceeded favorably. The child exhibited signs of having been dead several days.

The well established fact, that hæmaturia may be produced by absorption and inhalation of the vapors of turpentine; the occurrence of the urinary disorder several days previous to the miscarriage; the death of the fœtus at the time of the vesical irritation, and the absence of other appreciable causes, lead to the conclusion, that the cause assigned was the true one.

Dr. Miner remarked that there was a popular notion, that it was hazardous for a pregnant woman to remain in a room which had been newly painted, and he enquired whether the danger was due to the turpentine, or to the lead.

Dr. Rogers remarked, that painters often suffered from working in a close room, and were able to perceive the odor of the turpentine in their urine. This he had repeatedly observed.

Dr. Hawley mentioned that Wood cites, in his Practice of Medicine, a case of hæmaturia, produced by the absorption of turpentine.

Dr. Jansen detailed a fatal case of small-pox which had lately occurred in his practice. This case occurred three weeks ago. The patient was a a little boy eight years old, who had been ill two days before Dr. Jansen saw him. He was taken with the usual initiatory symptoms of variola, namely, chills, vomiting, headache, pains in the back and lions, and a slight rash had appeared on his forehead. The next day, this had extended to the wrists; on the third day there were punctuations, and on the fourth day it became confluent. The treatment was very simple at first, consisting merely of a Dover's powder occasionally, the bowels being kept open with castor oil. On the eighth day after the first appearance of the eruption, pustulation occurred; the pulse was then but 75. On the third day after pustulation, there was intense pruritus, which was relieved by the application of a decoction of bran and hops, with a little cherry bark. The case progressed for a few days, not unfavorably, but the patient suddenly died in the night, while the mother, who had been the constant attendant, was asleep. Dr. Jansen was at a loss for the immediate cause of death. The mother was afterwards attacked with the disease in a very mild form, only five or six pustules appearing on her person.

Prof. WHITE made some remarks on the precautions which should be taken by the practitioner, in order to prevent transportation, by himself, of the disease from one patient to another. When he had a case of variola under treatment, he was accustomed to remove the carpet from the room place the bed in the centre, and when he entered the house, to change his coat for one which he kept for the purpose, buttoning it tightly up to the throat. On entering the room of the patient, he was careful not to sit down; and, in that manner, the only parts of his person which could come in contact with the disease, were the soles of his feet, which were against the bare floor, and the tips of his fingers, with which he examined the pulse. He then changed his coat, washed his hands carefully, and took a short walk in the fresh air, before visiting another patient. He was not aware of ever having transported the contagion of small-pox, in twenty-five years of practice, when he had one or more cases every year.

Prof. White was of the opinion, that variola was not often transmitted by a physician, but he had always deemed it his duty to take these precautions while in attendance upon a case of the disease.

The subject of the transportability of small-pox was then discussed by Drs. Treat, Miner, Strong, Jansen, Rogers, Ring, Gould, Newman, and Boardman. Several interesting cases of this disease were described, where the probable origin had been traced. Dr. Treat mentioned one, which was inexplicable, until it was discovered that the victim had slept in a bed, and in the same bed-linen, which had been occupied by a person who had been recovered of small-pox for six weeks. He also cited the experiment, which is related in Thomas' Practice, where the small-pox matter was wrapped in cotton, and children, unprotected, were placed around a table on which it had been put; there resulted no infection. Dr. Treat had long been of the opinion, from his investigations into the history of this subject, that the disease could not be conveyed by emanations.

Dr. MINER mentioned an example of infection, which occurred at Cambridge, Mass., where fifty workmen were employed in excavating an old pest-house, which had not been used for twenty years; out of this number twenty cases of small-pox occurred. He himself had frequently been exposed to the contagion, but had taken the disease only when it had seemed impossible that it should be transmitted in any other way than by the air.

Dr. Strong mentioned an epidemic of small-pox, which had occurred in a country town in which he resided. This, apparently, was introduced by a stranger passing through, who was observed to have an eruption on his forehead. It first attacked a family in a house removed a half a mile from any other, and from thence resulted about forty-five cases. Thirty of these cases he had treated. He had used no precaution further than washing his hands after examining the patient, and had never communicated the disease.

Dr. Jansen had traced several cases of the disease in this city, from a German family, two of whom died of it; and it was probable that it was introduced there by a boy from the Ebenezer settlement, a few miles from the city.

Dr. Rogers mentioned a case where the exposure was very general, and undoubtedly to many unprotected persons, from a patient in whom the disease had been developed into the most virulent confluent form, before it was detected. This patient's room had been daily filled with visitors, not one of whom had contracted the disease.

Dr. Newman said that he had never carried the disease to any of his patients; that he had charge, at one time, of the pest-house, and had attended there two cases of labor. In the first case, he immediately vaccinated the new born infant, and succeeded in protecting it, the vaccination working admirably. In the other case he vaccinated the child immediately, but unsuccessfully, and revaccinated it at the end of three days, with success. Both these children were protected. He had doubts as to the transmission of the contagion by the atmosphere, and thinks that no cases have ever been traced to the pest-house in this city.

Dr. Ring remarked on the influence which temperature exerted upon the contagious material. He considered precautions more necessary to the physician in warm than in cold weather. Freezing destroyed the virulence of the matter. He therefore considered it prudent to change some of the clothes after visiting a small-pox patient in the summer, while it was not necessary in winter.

The general opinion of the gentlemen of the Association was, that while the contagion of small-pox was rarely transmitted by a medical attendant, yet it was barely posssible, and, therefore, it was the duty of every physician having cases under his care, to take reasonable precautions. In answer to the inquiry, as to the prevalence of the disease in the city of Buffalo, it was ascertained that cases were not as numerous as was generally supposed, and as had been asserted by the daily press. At that time there were only fifteen cases under treatment in the entire city.

Dr. GOULD presented a specimen of a piece of the alveolar process, which he had removed from a little girl, eight years old, who had been salivated by a homœopathist. This child had been treated for a fever by the homœopathic practitioner, and Dr. Gould had removed this piece of bone with the forceps, after the patient had passed out of his hands. He thought that it was a case of arsenical salivation.

Dr. TREAT mentioned a case, in which a needle had passed in at the metatarsal bone of the little toe, and out at the great toe, some weeks after. The needle had probably been received about three weeks before it was extracted. At the end of that time, it appeared beneath the skin, and was easily removed.

Dr. BOARDMAN had carried a needle in his own foot for three months. The parts had been opened, but the needle could not be extracted. At the end of three months, it was removed, and during that time had given him no more uneasiness than an occasional pricking.

Dr. WYCKOFF presented a beautiful specimen of a calculus, which had been discharged from the bladder of a female, at 40 years. She had before been healthy, and had bore eight children. She was seized with pain in the bladder, which continued for thirty-six hours, with hæmaturia. She then passed the calculus without much distress. The specimen was the size of a large bean, beautifully crystaline, and apparently consisted of oxalate of lime.

The Association then adjourned.

AUSTIN FLINT, JR., M. D.,
Secretary.

